# How levelling and scan line corrections ruin roughness measurement and how to prevent it

**DOI:** 10.1038/s41598-020-72171-8

**Published:** 2020-09-17

**Authors:** David Nečas, Miroslav Valtr, Petr Klapetek

**Affiliations:** 1 CEITEC, Masaryk University, Kamenice 5, 625 00 Brno, Czech Republic; 2 CEITEC, Brno University of Technology, Purkyňova 123, 612 00 Brno, Czech Republic; 3 Czech Metrology Institute, Okružní 31, 638 00 Brno, Czech Republic

**Keywords:** Characterization and analytical techniques, Imaging techniques, Scanning probe microscopy, Nanometrology, Statistics, Scientific data

## Abstract

Surface roughness plays an important role in various fields of nanoscience and nanotechnology. However, the present practices in roughness measurements, typically based on some Atomic Force Microscopy measurements for nanometric roughness or optical or mechanical profilometry for larger scale roughness significantly bias the results. Such biased values are present in nearly all the papers dealing with surface parameters, in the areas of nanotechnology, thin films or material science. Surface roughness, most typically root mean square value of irregularities Sq is often used parameter that is used to control the technologies or to link the surface properties with other material functionality. The error in estimated values depends on the ratio between scan size and roughness correlation length and on the way how the data are processed and can easily be larger than 10% without us noting anything suspicious. Here we present a survey of how large is the problem, detailed analysis of its nature and suggest methods to predict the error in roughness measurements and possibly to correct them. We also present a guidance for choosing suitable scan area during the measurement.

## Introduction

The impact of roughness on interaction of solid bodies with outer world is a long studied problem that can be traced at least to Leonardo da Vinci’s friction experiments^1^. Surface roughness is related to imperfections in nearly all manufacturing technologies^2–7^ and as such widely spread phenomenon it had to be addressed many times in past in relation to foundations of modern engineering. With the advent of smaller and smaller devices and their functional parts, the impact of roughness is only increasing^8–10^, and in the area of nanoscience and nanotechnology, where the typical sizes of objects are comparable to the roughness values naturally coming from different technologies, it can become dominant effect. Surface properties can be then affected more by its texture than by the material composition^11,12^. In order to get the correct surface function, surface roughness must be controlled, either to be minimal (e.g. for optical thin films, solar cell interfaces) or tuned to have certain statistical properties (e.g. in surface enhanced Raman Spectroscopy^13,14^, microfluidics^15,16^, plasmonics^17,18^ or cell adhesion and migration^19,20^). Roughness can also be an interesting phenomenon itself—studying its scaling during surface growth and determination of the universality class can provide insight into the physical processes involved^21–25^.

For all this roughness needs to be measured, which is on the nanoscale typically done using Scanning Probe Microscopy (SPM) techniques, such as Atomic Force Microscopy (AFM) and on large scale by a profilometric technique, either optical or tactile. Images of different roughness realizations, accompanied with simple statistical results, are therefore a common content of nanoscience and nanotechnology papers. Frequently, like in the case of electromagnetic field enhancement, the functional properties do not depend on roughness linearly and instead exhibit various exotic dependences, which makes roughness optimization much more challenging. In this work we show that the reported roughness results can be often wrong, which potentially affects also the conclusions taken from them and we suggest remedies to prevent this.

All measurement techniques have limitations and surface roughness is never measured in a statistically ideal way, which would mean infinite area with infinite resolution. In contact scanning methods such as AFM or profilometry the resolution is limited by finite probe size, in optical methods by finite wavelength. The measurement area is even more limited, usually by the measurement device construction, and often it is just a tiny fraction of the surface, even if multiple regions are measured to improve statistical representativeness. In contrast to method-specific artefacts, e.g. tip convolution in the contact methods^26^ that affect directly the statistical results across different scales^27,28^, effects related to finite area are universal. They would impact even ideal measurements in which the measured profile perfectly traces the surface topography.

Surface roughness is, conceptually, a statistical quantity^22,29^. Discussing roughness, we can think of an infinite ensemble of surfaces (possibly infinite themselves), usually corresponding formally to a random process, which may or may not be wide-sense stationary. Here we will treat the simplest case, which is roughness generated by stationary processes, and we will focus on the estimation of their parameters using a finite measurement of one of the realizations. In particular, we will study the consequences of finite measurement area and levelling/background subtraction as these are the most important and widespread deviations from ideal statistical treatment in practice.

One consequence of the finite scan size is that the estimated parameter, for example mean square roughness $\sigma$, estimated from a profile of length *L* by

1 $$\begin{aligned} {{\hat{\sigma }}}^2 = \frac{1}{L} \int _0^L z(x)^2 \,\mathrm {d}x \end{aligned}$$

is itself a random variable, as we denote with a hat. It has a dispersion, which is possibly large^22^. Note that expression (1) is equivalent to mean square roughness Rq for profiles^30,31^ and Sq for images^32^, as defined by the standards. From *N* discrete measured values $z_i$ ($i=1,2,\dots ,N$), Sq is usually realized by the sum

2 $$\begin{aligned} \mathrm {Sq} = \left[ \frac{1}{N} \sum _{i=1} z_i^2 \right] ^{1/2}\;. \end{aligned}$$

The most important problem is that this estimate is also biased. Heights *z*(*x*) entering (1) are levelled to have zero mean value. As the scan size is finite, this introduces bias, which is well known and discussed for correlated data in classical signal processing textbooks^22,33,34^. In a real world the situation is even worse as the subtraction of the mean value is rarely the only preprocessing applied to topographical data before roughness evaluation. Various instrument related imperfections usually need to be considered. Local defects, either coming from the feedback loop faults or unwanted surface contamination are often removed, although this may be unnecessary as roughness evaluation algorithms for irregular regions allow excluding arbitrary image parts from processing^35^. The misalignment between microscope axes and surface normal is removed by mean plane subtraction. Frequently also scanner bow is removed by higher order polynomial fitting^29^. Individual profiles misalignment related to mechanical and thermal drifts in the slow scan axis is corrected. Finally, roughness may have to be separated from sample form using frequency-space filtering, wavelet processing or subtraction of specific geometrical shapes such as sphere. All these methods are applied after the measurement, so if it is found that the roughness results are corrupted by them, it is usually late. The analysis and guidance provided in this paper will hopefully help to prevent such situation.

As could be guessed, the impact of levelling must somehow depend on characteristic lateral scale of the roughness and its ratio to the scan length *L*. Intuitively, if the scan is very long and the lateral scale of the roughness short, the estimated background will approach the true one and the bias will be minimal. As a measure of the lateral roughness properties we use the correlation length *T*, one of the common parameters quantifying the lateral scale of roughness, defined as the distance at which the autocorrelation function (ACF) decays to $1/\mathrm {e}$ of its maximum.

**Figure 1:**
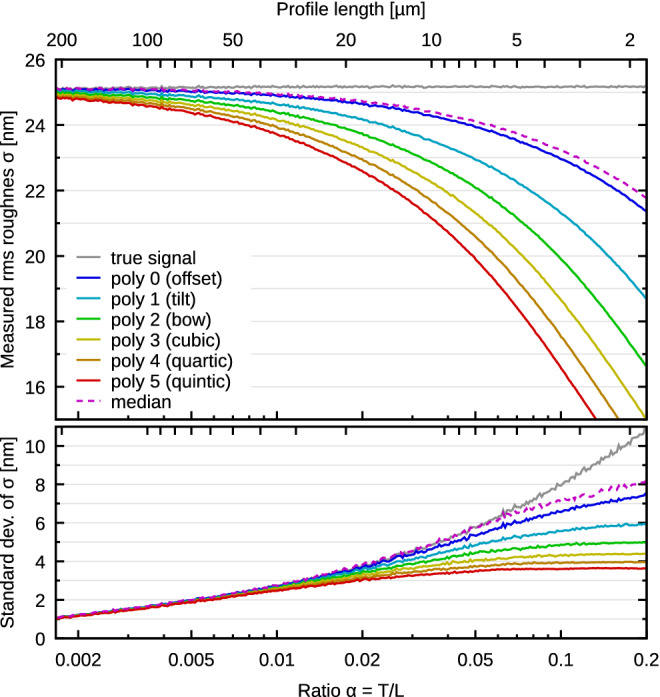
Measured roughness and its standard deviation as a function of profile length for a Simetrics reference sample, showing the bias evolution for shorter profile lengths and its dependence on the levelling.

For a real-world illustration of the impact of profile length on roughness quantities, we performed a set of long profile measurements of a randomly rough reference sample from Simetrics (ARS f3). The dependency of measured mean square roughness $\sigma$ on $\alpha =T/L$ is plotted in Fig. 1. The figure shows both the bias and standard deviation of measured $\sigma$ values. Both are quite large—and while the larger contribution to the uncertainty may come from the dispersion, this part can be reduced by repeated measurement (as is a good practice anyway). The bias, on the other hand, is inherently tied to *L* and repeated measurement is of no help in combatting it.

## The problem

The finite profile length, combined with levelling, is the key aspect of the problem. Ideally, one would take infinitely long profiles that would not need levelling, however this is practically not possible. Some of the steps used to remove background arise from measurement imperfections (scanner bow), some remove real base shape of the measured object. Often they remove both to some degree—and by intentionally removing certain degrees of freedom they also always remove inadvertently a part of the roughness. For instance in the case of the mean value, we subtract it because the measured surface height *h*(*x*) is not the roughness signal *z*(*x*). It is offset by some background, here a constant base height *B*:

3 $$\begin{aligned} h(x) = z(x) + B\;. \end{aligned}$$

The background *B* is non-random (at least from the roughness standpoint), but unknown. We estimate it as the mean value of the height because the expected value of *h* is $\mathrm {E}[h]=B$. However, the subtraction of this estimate removes not just *B* but also a part of the roughness.

**Figure 2:**
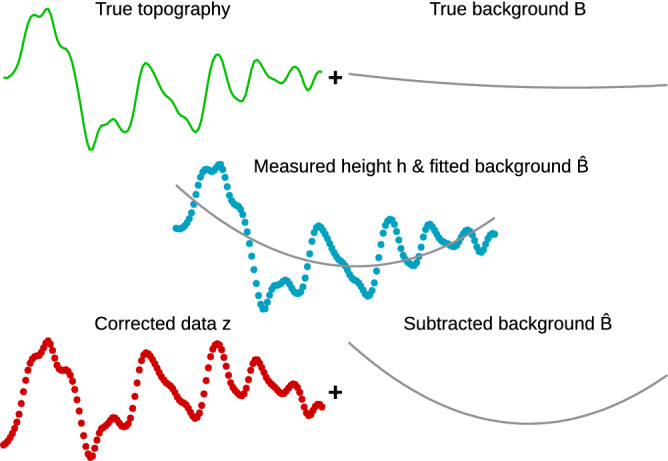
General scheme how background removal distorts the data.

Although the expected value of *z* is zero, the mean value of *z*(*x*) over a finite interval is a random variable, not zero—yet we make it zero anyway. The same principle—removing some part of roughness by levelling—applies also for higher order levelling, and is in fact even worse. This is illustrated in Fig. 2 for levelling by a second-order polynomial *B*. The true topography and true background (bow) combine to measured data. Background fitting is greedy and takes away not just the true background, but also roughness components which match it—randomly. The corrected data are then missing these components. The evaluated roughness parameters are therefore biased towards lower values and we will see later this bias can commonly be quite large.

As the bias is negative (we always remove some roughness) and proportional to $\sigma ^2$, it is convenient to introduce the relative bias $\beta$^36^. For the rest of the paper

4 $$\begin{aligned} {\mathrm{E}}[{\hat{\sigma }}^2] = \sigma ^2 (1 - \beta ) \end{aligned}$$

to simplify notation. If we know $\beta$, replacing ${\hat{\sigma }}^2$ with ${\hat{\sigma }}^2/(1-\beta )$ corrects the bias. Our goal is to estimate $\beta$ for different levelling approaches.

## Roughness measurement in the wild

We were curious how much real-world roughness results could be biased, or at least what are the typical *L*/*T* ratios—and how they compare to what various roughness measurement standards recommend. Starting from the second point, standards and roughness reference samples do come with recommendations for measurement length (or area), although interpreting them in terms of the ratio may not be straightforward.

ISO 19606^37^ recommends measuring 20–50 mean widths of profile elements (RSm). The relation between RSm and *T* depends on the roughness character. They are of the same order of magnitude, but for instance for Gaussian ACF the RSm is approximately $4\times$ longer, whereas for exponential ACF the two lengths are similar.

ASME B46.1^31^ recommendations translate to evaluation length of approximately $10 \times \text{RSm}$, but only for periodic surfaces which are not relevant here. For non-periodic surfaces the recommended length is based on the value of mean roughness Ra. The same holds for ISO 4288^38^, where evaluation length for non-periodic profiles is again based on amplitude parameters.

The evaluation procedures are more complex than discussed here, involving several different lengths that could be considered *L*. While the overall form is removed from the entire profile, for roughness parameter evaluation the profile is usually split to several sampling lengths, which are shorter. However, the upper limit of spatial frequencies included in roughness is given by the cut-off wavelength $\lambda _c$. Although it is not a perfect match, the question closest to the one considered here is ‘how roughness parameters depend on $\lambda _c/T$?’

For image data the procedures and parameters are given by the complex ISO 25178^32^. Spatial frequencies remaining in the evaluated surface depend on filtering (L- and S-filters) and form removal (F-operations), as defined by the standard, and the corresponding set of rules cannot be distilled into a simple *L*/*T* criterion. We only note that it allows, for instance, the application of F-operations with nesting index five times the scale of the roughest structures—which can translate to removal of relatively high frequencies.

Furthermore, even when AFM software evaluates roughness parameters according to a standard, measurement settings and procedures deviate in practice, often a lot. In fact, in fundamental science the instrument, sample and measurement method may all be unusual, idiosyncratic or unique. It would be unreasonable to insist on measuring roughness according to standards in such cases. However, it still should be measured correctly and accurately, given the constraints.

**Table 1:** Journals used for the ‘in the wild’ study.

Journal	2D	3D	Total	Papers
ACS Nano	5	4	9	5
Applied Surface Science	242	132	374	78
Electrochimica Acta	28	23	51	14
International Journal of Advanced Manufacturing Technology	17	64	81	18
Materials Research Express	89	86	175	41
Nanotechnology	42	13	55	14
Surface and Interface Analysis	59	51	110	25
Surface Topography: Metrology and Properties	79	38	117	17
Thin Solid Films	263	1	264	64
Ultramicroscopy	4	0	4	1
Wear	39	50	89	23

Columns 2D, 3D and total summarize how many images were extracted and processed.

This brings us back to the first point. Answering the question what *T*/*L* ratios actually occur in practice was in principle easier: it only required a sizeable sample of real-world data. We extracted it from literature, according to the following rules:

- Published in selected journals—see Table 1—in years 2017 and 2018.
- Surface roughness is quantified in the paper and it appears to be random.
- Topographical images are included in paper or supplementary information and allow determining *L* and estimating *T*.
- It is reasonable to assume that the presented images were used to measure roughness.

This resulted in a set of 1,629 images from 300 papers covering a range of scales, samples, techniques and journal impact factors (the full list of DOIs is in Supplementary material). Only image data were used because the extraction of profiles from 1D plots was found unreliable and prone to odd artefacts. Even reproductions of images in journals could be quite far from topographical AFM data; a description of how the results were obtained from the images in order to miminize the systematic errors is given in the Methods section.

**Figure 3:**
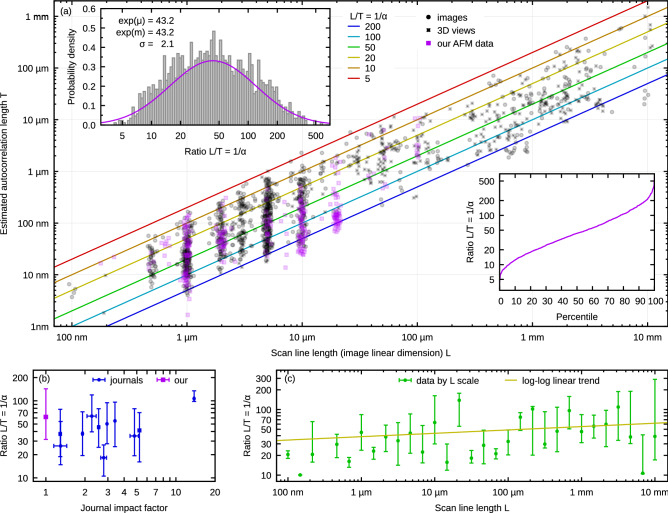
Ratio *L*/*T* in real-world roughness measurements: (**a**) overview of scan area size (along the fast axis) and correlation length of the surface; (**b**) ratio *L*/*T* as a function of journal impact factor (with our data added at 1.0); (**c**) ratio *L*/*T* as a function of scan line length *L*. Points and bars in (**b**) and (**c**) represent medians and upper and lower quartiles.

We augmented the extracted images by our own AFM data acquired for roughness measurements over the course of several years—300 topographical images in total. This included measurements of roughness of many different materials from the areas of optics, solar cells, microelectronics, materials science and surface metrology. The results are plotted in Fig. 3. Each point in Fig. 3a represents one image—distinguishing values extracted from 2D views, 3D views and our own topographical AFM data. Insets show the overall probability density of $\log (L/T)$, together with the log-normal distribution matching best the data, and the empirical distribution as a percentile plot.

The first observation is that the typical $L/T=1/\alpha$ range remains more or less the same over the several orders of magnitude of scale and different techniques. This can be partially explained by analogous measurement speed constraints in scanning techniques. The typical *L*/*T* ratio does not increase noticeably in the region where optical techniques become viable (see Fig. 3a). Nevertheless, a slight increase can be seen in the plot by *L* scale (Fig. 3c), with Pearson coefficient of 0.13 for $\log L$ vs. $\log (L/T)$.

Hence, the uniformity over scales probably also follows from experimenters choosing the measurement area to what ‘feels right’—and this factor is scale invariant. In the AFM domain, there is a strong tendency to make scans with nice rounded dimensions (1, 2, 5 or 10 micrometres), whereas at larger scales this is less noticeable. A small random scatter was added to *L* in the plot to improve visualization of the high-density clusters.

An inset in Fig. 3a shows the overall distribution of *L*/*T* in logarithmic scale. The empirical distribution is likely distorted at both ends. Ratios around 5 and smaller are difficult to observe. The scanned areas become too small to carry information about true *T*, which is then underestimated based on spatial frequencies actually represented in the image. Conversely, high ratios require high pixel resolutions. The quality of reproduction in the journals could thus lower estimated ratios if they lie in the hundreds range. Anyway, the data can be modelled by a wide log-normal distribution centred around $L/T\approx 43$, with ratios below 20 (and over 100) still quite common.

Finally, we remark that the goal of this study was to get an overview, not to criticize published works. No correlation can be seen in Fig. 3b between journal impact factor and *L*/*T* (disregarding one outlier which had too few relevant papers). There are valid reasons why we measure with small *L*/*T* (large $\alpha$). Experimental constraints can prevent combination of measurements at different scales, which is otherwise a sound strategy how to combat frequency cut-offs^39,40^. One common reason is also consistency when studying roughness whose properties vary with external factors^41,42^. In this case usually *L* remains constant, but *T* varies. The variation can be dramatic, for instance when studying a dynamic roughening process^22–25,43^, resulting inevitably in both large and small *L*/*T* (note that in Ref.^43^ the bias was corrected). Yet this is also one of the cases where the bias can be the most problematic as it changes during the roughening, skewing the measured exponents.

As we will see, choosing *L*/*T* so large that the bias is consistently negligible is a demanding measurement strategy. The required *L* tends to be exceedingly long. Instead, we should choose a reasonable *L*/*T*, then estimate and deal with the bias.

## How data processing skews roughness results

Numerous algorithms are available for the processing of SPM data after measurement and a large class of them deals with surface levelling in some sense. As mentioned in “Introduction”, there are several reasons why data coming from a scanning technique need to be levelled:

- Instruments are typically constructed to measure flat samples. However, this does not mean that the *z* axis of the instrument is exactly perpendicular to the sample surface. In most systems this misalignment is less than $5\,^\circ$^44^. If the *z* axis is levelled manually by users much larger misalignment can be easily introduced. Even when the *z*-axis direction is preserved by instrument construction, a misalignment of about $0.1\,^\circ$, as practically observed e.g. for Dimension Icon by Bruker, still requires a correction. Otherwise the tilt would increase roughness measured on scan area of 100 μm by 50 nm.
- Some scanning instruments, typically AFMs, are relatively slow. The presence of thermal and mechanical drifts in the *z* axis, which is typically by construction the least symmetric one, leads to an apparent sample tilt. The drift rates of the best instruments are in nanometres per hour. On the other hand, in non-optimal conditions or devices not optimized for drift they can easily be the in order of tens of nanometres per minute^45^. Fine scans of roughness on large areas can take tens of minutes, so the effect is not negligible on any device. Moreover, the drift tends to be non-linear in time, requiring a higher order levelling.
- Low frequency noise can lead to shifts between individual scan lines that are used to construct the image; a similar effect can also be attributed to random drift in the *z* axis^44^. This can be exacerbated by the use of a mechanical contact in the microscope feedback loop, e.g. in AFM or contact profilometry. We can then observe the impact of scanning direction, which includes friction and/or elastic probe response. The effect is not deeply studied and even when the data come from the same scanning direction and the imaging conditions are excellent, they often do not match perfectly.
- Some scanning systems exhibit inherent non-flat backgrounds and can produce bow or other background shapes even for a flat sample. The background shape can be evaluated and removed from the measurement. This again means fitting and levelling (usually by polynomials), unless a complex analysis of the scanner properties is done^46^.
- Further levelling is needed if the sample itself is not flat and its form has to be removed prior to analysis. This is common when surface roughness is measured on fibres, rods or particles.

The choice of a particular data preprocessing algorithm is usually on the instrument user. This can lead to significant influence of the human factor and affect the results by errors in the order of several percent^47^. The variability of pre-processing methods used in practice is also why we are addressing the different types of levelling and estimate for them the roughness bias. Moreover, depending on the software and settings, plane levelling or row alignment (flattening) can be applied automatically during file opening or roughness evaluation, possibly even unnoticed by the user. This does not help understanding of the data processing and makes more difficult to take into account the effect of preprocessing on degrees of freedom or removal of spatial frequencies from the images.

It is also important to note that the choice of data processing steps could be optimized for the particular statistical properties acquisition, which is usually not done. It is common to process AFM image data row by row because roughness properties can often be determined more reliably in the direction of the fast scanning axis^22,29,35,48^. This means that levelling is applied to individual image rows instead of (or in addition to) the entire image. The results for individual rows then may be summed or averaged. The result for each row is biased as if we processed 1D data, not 2D. The same holds if any row-wise preprocessing is applied, such as removal of mean value from each individual row. It is, therefore, quite rare that the bias corresponds to the pure 2D processing case, even for image data.

For the elementary case of mean value subtraction from correlated data, the resulting bias is well known and noted in classic signal processing textbooks^22,33,34,49^. If the square of root mean square roughness $\sigma$ or Rq is estimated from heights *z* in a finite region [0, *L*] as follows

5 $$\begin{aligned} {\hat{\sigma }}^2 = \frac{1}{L} \int _0^L [z(x) - {\hat{\mu }}]^2 \,\mathrm {d}x\;, \end{aligned}$$

where the mean value is estimated

6 $$\begin{aligned} {\hat{\mu }} = \frac{1}{L} \int _0^L z(x) \,\mathrm {d}x\;, \end{aligned}$$

then its expected value is

7 $$\begin{aligned} {\mathrm{E}}[{\hat{\sigma }}^2] = \sigma ^2 - 2 \int _0^1 (1-t) G(Lt) \,\mathrm {d}t\;, \end{aligned}$$

where the second term gives $\beta$ by comparing with the definition (4). Symbol *G* denotes the autocorrelation function (ACF) of the signal (*t* is a scaled unitless distance). This result can be extended to images and higher-dimensional data. For common ACF types we obtain^22,36^

8 $$\begin{aligned} \beta \sim c\alpha ^D\;, \end{aligned}$$

where *D* denotes the dimension (1 for profiles, 2 for images, etc.). Numerical factor *c* is of the order of unity and depends on the ACF type. In other words, the relative systematic error of ${\hat{\sigma }}^2$ due to finite-area bias behaves approximately as $\alpha$ and $\alpha ^2$ for 1D and 2D data, respectively.

The systematic error does *not* depend directly on the number of measured values *N* (provided it is sufficiently large). Increasing *N* without making the measurement area larger is of no help and Bessel’s correction (replacing 1/*N* with $1/(N-1)$ when estimating variance) is ineffective. For correlated data the correction factor is not $1-1/N$, but approximately $1-c\alpha ^D$.

### Polynomial levelling

The most common background removal method in roughness measurement is polynomial levelling, i.e. subtraction of polynomials, which includes the subtraction of a mean plane. It is a linear method—the polynomials are fitted to the data using linear least squares. We note that not all background removal methods are linear—for instance the subtraction of median or a fitted spherical surface is non-linear, but many common ones are.

The procedure for estimation of bias originating from mean value subtraction can be extended also to these cases. Expression (7) then becomes

9 $$\begin{aligned} {\mathrm {E}}[{\hat{\sigma }}^2] = \sigma ^2 - 2^D \int _0^1 C(t) G(tL) \,\mathrm {d}t\;. \end{aligned}$$

Function *C*(*t*), which replaced $1-t$ in (7), characterizes the autocorrelations in the linear space spanned by the set of basis fitting functions^36^—for instance powers in the case of polynomial background or sines and cosines in frequency-space filtering.

To obtain the relative bias $\beta$ for one-dimensional polynomial background we need to specify the form of the ACF. Two common models are Gaussian and exponential^22,29^

10 $${G^{{\rm{Gauss}}}}\left( x \right) = {\sigma ^2}\exp \left( { - {x^2}/{T^2}} \right)\;{\rm{and}}\;\;{G^{{\rm{exp}}}}\left( x \right) = {\sigma ^2}\exp \left( { - \left| x \right|/T} \right).$$

**Figure 4:**
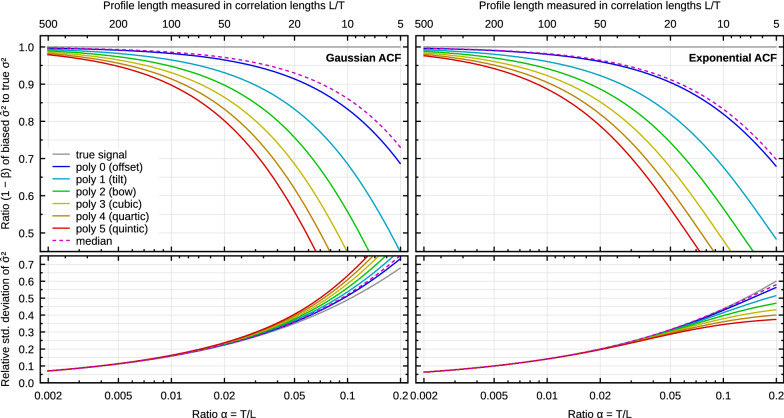
Numerical results for polynomial background removal from profiles with Gaussian and exponential ACFs. Upper: Bias, plotted as measured ${\hat{\sigma }}^2$ divided by true $\sigma ^2$ for several polynomial degrees. Lower: Standard deviation of measured ${\hat{\sigma }}^2$ (for one evaluation length), relative to the value.

Although closed-form expressions for $\beta$ exists in the case of polynomial levelling^36^, usually the leading-order approximations for small $\alpha$ are sufficient. In one dimension these are

11 $$\beta _j^{{\rm{Gauss}}}\sim \left( {j + 1} \right)\alpha \left[ {\sqrt \pi - \left( {j + 1} \right)\alpha } \right]\;{\rm{and}}\;\beta _j^{{\rm{exp}}}\sim 2\left( {j + 1} \right)\alpha \left[ {1 - \left( {j + 1} \right)\alpha } \right],$$

where *j* denotes the degree of the polynomial. The systematic error of ${\hat{\sigma }}^2$ corresponding to these two ACF types is plotted in Fig. 4 as a function of scan line length.

Although a careful comparison of bias plots in Fig. 4 reveals some differences, the curves for Gaussian and exponential ACF are remarkably similar. The leading terms in (11) differ by factor $\sqrt{\pi }/2\approx 0.886$, which is relatively close to unity. However, this ratio applies when both corrections are small. The curves are actually even closer for larger $\alpha$ thanks to the higher order terms.

For both ACFs the bias is quite high—at least a few percent even for *L*/*T* in the range of hundreds. When we consider the median ratio $L/T\approx 45$ found in the wild, the bias is approximately 8% for tilt removal. It becomes 25% for rather ordinary bow removal with $L/T=20$. Finding more extreme yet still realistic examples is not difficult, for instance 3rd order polynomial removal with $L/T=15$, for which the bias becomes approximately 40%.

For reference, the plots also include standard deviations of ${\hat{\sigma }}^2$. The variance of ${\hat{\sigma }}^2$ exhibits the same general behaviour as the bias, i.e. it is proportional to $\alpha ^D$. Leading-order asymptotic expressions are known^22^. Curves in Fig. 4 were obtained numerically in order to illustrate the full non-linear behaviour. Nevertheless, they more or less all coincide for small $\alpha$, matching the asymptotic expressions. A dependence on the polynomial degree only starts to appear for large values.

**Figure 5:**
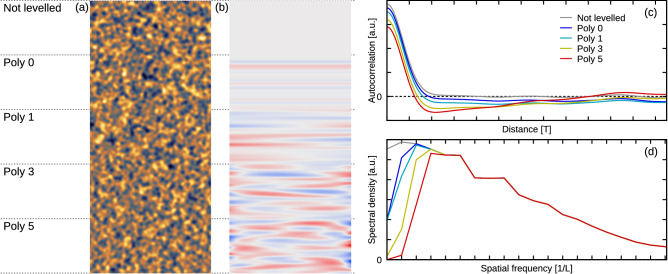
The effect of polynomial levelling of scan lines on topographical images: (**a**) surface topography after the application of levelling; (**b**) removed line polynomial background (blue and red indicate negative and positive values, respectively); (**c**) one-dimensional row-wise ACF; (**d**) spectral density of spatial frequencies.

Although the effect of levelling on statistical parameters can be dramatic, in a visual inspection of topographical images it is barely noticeable, as illustrated in Fig. 5a. The single topographical image (artificially generated with Gaussian ACF and $\alpha =0.025$) in fact consists of five blocks. In each block the rows were levelled differently as denoted.

It is possible to discern the slight deformation present mainly close to image boundaries, in particular for the fifth degree polynomial levelling. However, it requires a careful inspection; even if the removed background is quite different for individual blocks, as seen in Fig. 5b (which has a false colour range comparable to Fig. 5a). On the other hand, plots of one-dimensional ACF and spectral density are clearly affected as illustrated in Fig. 5c,d. We can see the region of negative values developing around the maximum in ACF—while higher degree levelling erases larger and larger portions of the spectral density at low frequencies.

Figure 5 also helps with intuitive understanding why the bias is proportional to $\alpha =T/L$ instead of 1/*N*. Spectral density is substantially non-zero only in a region of width approximately $1/(\alpha L)$ around the zero frequency. Furthermore, it usually attains the largest values at the lowest frequencies. Therefore, even if a single spatial frequency is removed (or suppressed), it erases a disproportionally large part of the roughness.

**Figure 6:**
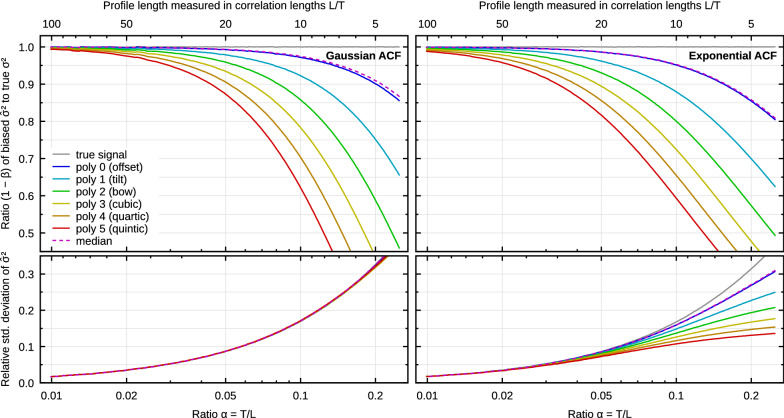
Numerical results for 2D polynomial background removal from image with Gaussian and exponential ACFs. Upper: Bias, plotted as measured ${\hat{\sigma }}^2$ divided by true $\sigma ^2$ for several polynomial degrees. Lower: Standard deviation of measured ${\hat{\sigma }}^2$ (for one image), relative to the value.

### Other data levelling methods

The approach can be applied to other background removal procedures. An important case is two-dimensional polynomial levelling. For Gaussian and exponential ACFs and total degree of the polynomial *j*, the leading terms for small $\alpha$ are^36^

12 $$\beta _j^{{\rm{Gauss}}}\sim \frac{{\pi \left( {j + 1} \right)\left( {j + 2} \right)}}{2}{\alpha ^2}\;{\rm{and}}\;\beta _j^{{\rm{exp}}}\sim \pi \left( {j + 1} \right)\left( {j + 2} \right){\alpha ^2}.$$

The bias and standard deviation of ${\hat{\sigma }}^2$ are plotted in Fig. 6 for these two basic ACF types (including higher order terms). As in the 1D case, the results for exponential ACF are relatively similar, although the factor between the leading terms is now 1/2 and the curve shapes differ more noticeably.

Since the leading term is proportional to $\alpha ^2$, the bias is much smaller than for profiles, in particular for small $\alpha$. When comparing Figs. 4 and 6, notice the different ranges of $\alpha$ which are [0.002, 0.2] and [0.01, 0.25], respectively. Even though the curves become steeper for 2D background removal when $\alpha$ is large, the bias remains small compared to 1D for all reasonable image dimensions. Therefore, when both 1D and 2D preprocessing is applied to image data, the bias caused by 2D preprocessing is usually negligible. More so, since the 1D levelling already removes some degrees of freedom the 2D levelling would.

Figures 4 and 6 also include results for median levelling, i.e. subtraction of the median. Median subtraction is available in some AFM software^50^ as an alternative to mean value subtraction because median is less sensitive to outliers. Median subtraction is a non-linear operation and thus outside the framework for background subtraction by linear fitting. Therefore, the results in Figs. 4 and 6 were obtained numerically—and they show a behaviour very similar to mean value subtraction. Although the two operations differ markedly with respect to outliers, from the bias standpoint they both essentially remove one degree of freedom.

**Figure 7:**
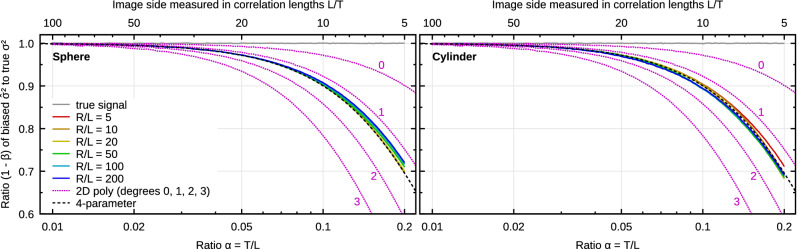
Numerical results for removal of spherical and cylindrical backgrounds from Gaussian randomly rough surfaces.

As the final levelling method we consider non-linear form removal by subtracting spherical and cylindrical surfaces fitted to the data. Removal of a base spherical or cylindrical shape is a common levelling operation. Sphere or cylinder fitting is non-linear, whether implemented by ordinary non-linear least squares by means of Levenberg–Marquardt algorithm^51^, or total least squares, i.e. using orthogonal regression^52^. However, in a small region of parameter space close to the optimum, the problem can be linearized. In principle, this allows constructing a function *C*(*t*) characterizing the corresponding linear function subspace and using it in (9) to estimate the bias $\beta$.

Or, instead, we can just imagine carrying out all these tedious steps. If the non-linear model has *p* parameters, the linearized version will also have *p* parameters. If the form is smooth its subtraction removes the lowest spatial frequencies similarly to polynomial levelling. We can thus expect that the non-linear background removal will behave similarly to linear background removal with the same number of free parameters. The bias will be likely somewhat smaller because the shape is constrained in a manner making it less effective at removal of low spatial frequencies.

The upshot is that from the bias standpoint, the subtraction of the base shape should be similar to the subtraction of a polynomial background with a matching number of free parameters. Both spherical and cylindrical surfaces are described by four parameters. For spheres we can choose radius and three coordinates of the centre—and radius, orientation, height and one horizontal coordinate for cylinders. Low-order 2D polynomials with limited total degree have 1, 3, 6, 10, etc. coefficients (triangular numbers). So there is no direct match. However, expressions such as (11) and (12) suggest approximately substituting

13 $$\begin{aligned} {j_{1{\text{D}}}} = p-1 \quad \text {and}\quad {j_{2{\text{D}}}} = \bigl (\sqrt{8p+1}-3\bigr )/2\;, \end{aligned}$$

as an effective polynomial degree *j* in 1D and 2D, where *p* is the number of free parameters. The 2D effective degree ${j_{2{\text{D}}}}$ is not necessarily an integer. However, the formulae (12) do not require integral *j*.

The efficacy of this estimate is illustrated in Fig. 7 for the removal of 2D spherical or cylindrical background and Gaussian randomly rough surfaces. The artificially generated surfaces had correlation length $T=30$ pixels and $\sigma =1$ pixel (heights must be the same physical quantity as lateral coordinates, otherwise spheres are ill-defined). The $\alpha$ ratio was varied as above and the ratio *R*/*L* of radius *R* to image linear dimension *L* was varied as well from 5 to 200.

The curves for all *R*/*L* ratios and both shapes are almost indistinguishable (forming narrow rainbow bands), confirming that the ratio of radius to image size can be disregarded in an approximate estimate (*R*/*L* does not enter (13) at all). Furthermore, they indeed correspond quite well to the simple counting estimate obtained for $p=4$, denoted ‘4-parameter’. For comparison the figure also includes curves for subtraction of 2D polynomial backgrounds of degree 0–3.

## Simple estimation and guidance

Graphs 4 and 6 can be used for the estimation of the bias and standard deviation of measured roughness (characterized by ${\hat{\sigma }}^2$) for Gaussian and exponential ACF. After estimating the correlation length *T*, one can simply divide it by profile or scan line length *L* to obtain $\alpha$ and look up the corresponding curve in the plot, according to the fitted polynomial degree.

However, considering the results in their entirety together with results for other cases^36^, we can distil an even much simpler estimate for mean square or average roughness. Although it is coarse and must never be used for correction, it is sufficient for judging the finite-area bias magnitude and a subsequent update of systematic uncertainties, conclusion that bias is insignificant or decision to measure again with different settings.

The procedure can be formulated as follows:

1. See whether you deal with the 1D or 2D case. When image data are processed row by row, it is 1D (profiles along the fast scanning axis).
2. Combination of 1D and 2D usually means you only need to care about the 1D part.
3. Estimate the correlation length *T* (from ACF or by other means, e.g. using spectral density).
4. Calculate *T*/*L* (1D; *L* is profile length) or $T^2/A$ (2D; *A* is image area).
5. Multiply it by the number of degrees of freedom removed. This means number of terms for polynomial (which differs from the degree, especially in 2D), or more generally number of fitting parameters.

The result roughly estimates the *relative* negative bias of either mean square or average roughness (ratio of bias and the roughness value). As an example consider $10\times 10\mathrm \,\upmu \text{m}^2$ scan size, scan line levelling using 2nd order polynomials (i.e. 1D case) and correlation length $T\approx 0.4\mathrm \,\upmu \text{m}$. In this case roughness will be underestimated approximately by $0.4/10\times (2+1)=12\,\%$.

If the simple estimate is considerably larger than 1/10, it is probably off due to non-linearity, but then the data were not worth much (for roughness measurement) to begin with. In such case it is necessary to repeat the measurement with larger scan line length to decrease *T*/*L*. A reasonable *L* can be found using the same method. A tool facilitating the estimation is now being developed for Gwyddion.

## Other parameters

So far the focus has been on the mean square roughness. It is an important parameter and it may be the easiest one to analyze mathematically, but it is just one of many roughness characteristics. Average roughness is often used instead of mean square roughness. Other common parameters involve higher moments of the height distribution, for instance skewness and kurtosis. Several are based on minima and maxima. Hybrid parameters are computed from local slopes or lengths and surface areas. Do they behave the same as $\sigma ^2$? If they do not, what makes them different?

**Figure 8:**
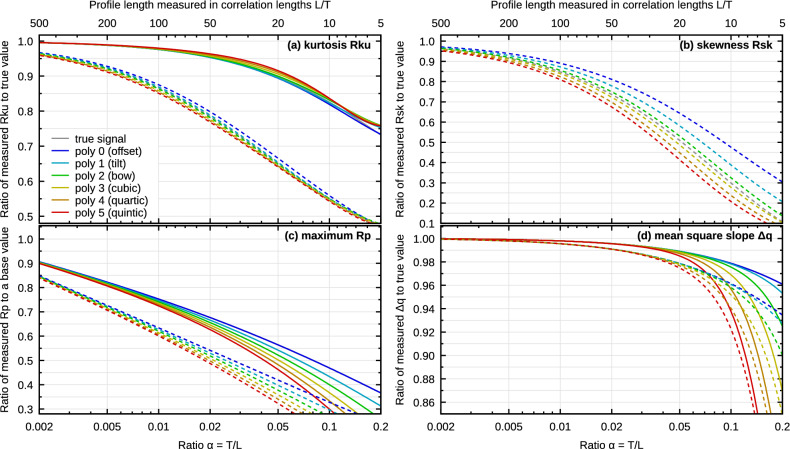
The effect of finite profile length and levelling on selected roughness parameters. Full and dashed curves correspond to values for symmetrical and skewed height distributions, respectively.

Levelling suppresses the highest and lowest values, in particular when they are clumped together. If a portion of a measured profile is consistently higher or lower than the rest, levelling attempts to pull this portion closer to the mean. For mean value or plane subtraction this effect is spread out over the entire data, but higher polynomial degrees are able to act more and more locally. And if the profile is shorter, they are more likely to suppress a part of roughness. Therefore, we can expect that plotting different roughness parameters as functions of $\alpha$ will again produce curves resembling Figs. 1, 4, 6 and 7. Although this is mostly true, there are also notable deviations. The following list briefly summarizes the behaviour of several common roughness parameters (defined by the 1D^30,31^ and 2D^32^ roughness standards), based on numerical results. Selected dependencies are also plotted in Fig. 8 for 1D data, Gaussian ACF and two height distributions, symmetrical Gaussian and skewed with skewness of 1.

- All simple quantities characterizing the vertical scale of roughness behave like ${\hat{\sigma }}$. This includes mean square roughness Rq and Sq and average roughness Ra and Sa. This is because for a fixed distribution of heights they are all mutually proportional. Even though levelling alters the height distribution slightly, this effect tends to be negligible^36^.
- Kurtosis (Rku and Sku) also behaves qualitatively similarly (Fig. 8a), with one important difference. Its bias almost does not depend on levelling. In particular, kurtosis is underestimated even without any levelling at all. This is because it is calculated as the ratio $\mu _4/\sigma ^4$, where $\mu _4$ is the fourth central moment of height distribution. Both ${\hat{\mu }}_4$ and ${\hat{\sigma }}^2$ have correct mean values for the true signal. However, division is a non-linear operation and ${\hat{\mu }}_4$ and ${\hat{\sigma }}^2$ have large variances (see Fig. 4). The mean value of the ratio is, therefore, skewed according to the Jensen’s inequality^34,53^. However, this is still not the full explanation because we would then expect kurtosis to be overestimated, not underestimated. Underestimation arises because ${\hat{\mu }}_4$ and ${\hat{\sigma }}^2$ are also strongly correlated (and their distributions are not symmetrical).
- Everything said about kurtosis also holds for skewness (Rsk and Ssk). Additionally, we must note that levelling tends to make the height distribution more symmetrical. Therefore, skewness is reduced in absolute value with increasing $\alpha$ (Fig. 8b). For symmetrical height distributions with zero skewness the skewness is unaffected and stays at zero.
- Minimum and maximum (and the various quantities derived from them) also exhibit specific dependencies on $\alpha$ that are not primarily caused by levelling (Fig. 8c). The maximum of *N* independent identically distributed random variables grows monotonically with *N*^34^. This still holds even for correlated data (the growth changes only quantitatively) and this effect dominates effects originating from levelling. Therefore, parameters based on minima and maxima are inherently incomparable between measurements with different $\alpha$. The limits for $\alpha \rightarrow 0$ are not even finite for common model distributions, so the figure shows ratio to a more or less arbitrary base value corresponding to a long base profile.
- Mean square slope $\Delta$q is largely unaffected by levelling (Fig. 8d). The reason is that levelling suppresses the lowest spatial frequencies. They contribute significantly to Rq because the spectral density *W*(*k*) has normally a maximum at zero. However, the lowest spatial frequencies contribute negligibly to $\Delta$q because $(\Delta \mathrm {q})^2\sim \int k^2 W(k)\,\mathrm {d}k$ and the factor $k^2$ already suppresses the lowest frequencies *k*.
- The same argument can be made for average slope $\Delta$a, developed profile length, surface area and similar quantities. They are all sensitive to high-frequency noise, but not to changes in the lowest spatial frequencies.
- Spatial parameters characterising lateral sizes of features are decreasing with increasing $\alpha$ as low spatial frequencies disappear from the data. Parameters such a correlation length behave similarly to ${\hat{\sigma }}^2$ (see also Fig. 5 which illustrates the narrowing of the autocorrelation function with increasing polynomial degree). However, for parameters based on feature counting and other concepts difficult to analyze mathematically, probably only a case-by-case numerical simulation would reveal their behaviour.

Even though the above list can seem like a long enumeration of peculiarities and exceptions, there are some common patterns. For parameters which depend on $\alpha$, the dependence starts to be significant in a similar range of $\alpha$ values. If we judge the bias of ${\hat{\sigma }}^2$ for specific $\alpha$ acceptable (or unacceptable), it is likely, albeit not guaranteed, that we would come to the same conclusion for other parameters.

## Conclusion

Roughness measurements results by Scanning Probe Microscopy methods can be, and in practice mostly are, significantly underestimated by measuring on areas that are not sufficiently large compared to the roughness correlation length. It is important to have this in mind when controlling roughness in a nanofabrication process or while comparing SPM values to other techniques. This bias originates from levelling routines that are used to preprocess the data and that are needed due to scanning methods imperfections. Even though such effect is, in its simplest case of mean value subtraction, well known in signal processing context, it is almost never taken into account in the context of nanoscale and microscale topography measurements. Moreover, the classic results do not cover the more complex SPM data preprocessing.

The estimated roughness then depends on the measured area size because the bias is a function of the ratio of the image size to the roughness correlation length. On the basis of a large survey of published images we found that this ratio is in practice typically around 40—however, its distribution is quite wide. Roughness then can be underestimated by 5–30%, depending on the levelling method. We can therefore see that there is a significant conceptual error in the generally used methodology for roughness measurements. Conclusions about more complex surface properties related to roughness (e.g. plasmonic enhancement or growth scaling exponents) should be therefore made carefully.

Having in mind the systematic errors in our roughness results is a first step to improvement. If we know which levelling method was used and make some assumption about the roughness correlation function shape, the error can be estimated. Using the estimates provided in this paper we can quantify how much the results are corrupted and either correct the scan area in subsequent measurements, include this to the measurement uncertainty, or correct the measured value. For this a simple guidance is provided.

Although the presented methodology was developed namely for the case of SPM or profilometry measurements the approach is suitable for most of the contact and non-contact scanning techniques. Moreover, apart from topography measurements, in principle it should be applied whenever a scanning technique is used to determine variance or other statistical properties of a physical quantity.

## Methods

### Experimental measurements

The measurement was done by the Nanopositioning and Nanomeasuring Machine NMM-1^54^ from SIOS company that, combined with a custom built AFM head, can be used for measurements over even centimetre areas. Contact mode was used with PPP-CONTR cantilevers (Nanosensors) and the probe-sample force was kept below 20 nN. Very slow scan speed (1 μm/s), selected on basis of repeated profile measurements tests was used to minimize the risk of tip degradation while scanning across rough sample. Polynomial levelling (with different parameters) was used to pre-process the data. Measurements were performed with millimetre long profiles (22 in total), at least 3000$\times$ longer than the estimated correlation length, and the sampling step was 3 nm. Data corresponding to shorter evaluation lengths were then obtained by cutting short segments from these base profiles. As many independent segments as possible were cut from each base profile until it was exhausted. The results for all segments were then processed to obtain the mean values and standard deviations. The abscissa step used to calculate the segment length dependencies was 64/63. The nominal roughness for the Simetrics ARS f3 sample as provided by manufacturer is $\text{Sa}=20\,\text{nm}$.

### Literature data processing

Raster pictures embedded in PDF documents were extracted and cut to only the greyscale or pseudo-colour mapped topographical image. The dimensions of the measured area were noted manually to cover the wide range of styles how this information is presented in papers. We dealt with transformations and artefacts as follows:

- pseudo-colour mapping: approximately undone using the most suitable from a few selected functions mapping RGB colour values to real numbers,
- inset boxes: cutting the image to a smaller vertical size (horizontal size *L* had to be preserved),
- large markings: data replacement using Laplace interpolation in Gwyddion^50^,
- 3D display: approximately undone by applying inverse perspective transformation using handle transform tool in GIMP^55^, and
- JPEG compression artefacts, interpolation, shading, small markings: disregarded.

Based on tests with images with known true *T*, this treatment was found sufficient for recovering its reasonable estimate. Fortunately, characteristic lateral scale of features is more resilient to transformations than the distribution of heights (for instance). The main exception is 3D display, where several competing effects can cause either under- or overestimation of *T* and its uncertainty was thus higher. When both pseudo-colour image and 3D view were shown for the same data in a paper, only the former was used.

### Numerical computations

For the spherical and cylindrical background removal simulations, images $15{,}000\times 15{,}000$ pixels were generated using Gwyddion^50^ *Spectral* data synthesis module with prescribed $\sigma$ of 1 pixel and Gaussian ACF. The correlation length was fixed to $T=20$ pixels while the *L*/*T* and *R*/*T* ratios varied. In total 10 independent images were generated. Smaller images were obtained by cutting independent rectangular regions of these large base images until the base image was exhausted. Centred spherical and cylindrical backgrounds, constructed analytically, were then first superimposed on the cut images and subsequently fitted by non-linear least-squares fitting using the Levenberg–Marquardt algorithm (as implemented in Gwyddion), and then subtracted. Initial parameter estimates for the fit were chosen as the true parameters (differing from the actually fitted optimum), mainly to improve the computation speed and avoid misestimation artefacts.

For the simulations of $\alpha$-dependencies of other roughness parameters, images $80{,}000\times 3{,}200$ pixels were generated again using the spectral method. Each image consisted of a set independent horizontal profiles with $\sigma$ of 1 pixel, Gaussian ACF and correlation length $T=30$ pixels and each profile was processed independently. Skewed value distribution was achieved by a postprocessing transformation $z\rightarrow [\exp (cz)-1]/c$ for $z\ge 0$ and $z\rightarrow -\ln (1-cz)/c$ for $z<0$, and normalization, with $c\approx 0.3272$ corresponding to skewness of 1. Shorter segments were cut from the base profiles until the profile was exhausted. Third and fourth central moment and parameters Rq, Ra, Rsk, Rku, Rp, Rv, $\Delta$q and $\Delta$a were evaluated for each segment and statistically processed.

## Supplementary information

Supplementary Information 1.

Supplementary Information 2.

## Data Availability

Literature images used for the ‘in the wild’ study are copyrighted and cannot be made available by the authors. The list of DOIs of papers from which they were extracted is available as Supplementary material.
