# Supplementary material for: How levelling and scan line corrections ruin roughness measurement and how to prevent it
Source: Sci Rep. 2020 Sep 17;10:15294. doi: 10.1038/s41598-020-72171-8 (PMC7499267; doi:10.1038/s41598-020-72171-8)
Supplement: Supplementary file 1 — Supplementary Information 1. [file 41598_2020_72171_MOESM1_ESM.pdf]

How levelling and scan line corrections ruin roughness measurement and how to prevent it — Supplementary information

David Nečas, Miroslav Valtr and Petr Klapetek

S1 — List of DOIs

The list is in a separate file `S1-doi-list.csv`.
